# Case Report: Hereditary ATTR-CA presenting with recurrent severe abdominal cramps: a diagnostic challenge

**DOI:** 10.3389/fcvm.2026.1879549

**Published:** 2026-09-08

**Authors:** Chunhua Mo, Kangla Liao, Yuanzhu Li, Suxin Luo, Bi Huang

**Affiliations:** 1 Department of Cardiology, the First Affiliated Hospital of Chongqing Medical University, Chongqing, China; 2 Department of Cardiology, the First Affiliated Hospital of Chongqing Medical University, Chongqing, China

**Keywords:** abdominal pain, left ventricular hypertrophy, multidisciplinary team, p.Ser43Asn, transthyretin cardiac amyloidosis

## Abstract

**Background:**

Hereditary transthyretin cardiac amyloidosis (ATTR-CA) is a rare autosomal dominant systemic disorder characterized by amyloid deposition in the peripheral nerves, myocardium, kidneys, and gastrointestinal tract. The clinical manifestations of ATTR-CA are nonspecific, resulting in high rates of misdiagnosis and underdiagnosis.

**Case presentation:**

We report the case of a 58-year-old woman who presented with recurrent severe abdominal pain and echocardiographic evidence of ventricular hypertrophy. Initial cardiac magnetic resonance imaging at an outside institution suggested left ventricular non-compaction (LVNC). Prior to receiving the correct diagnosis, she was incorrectly diagnosed with chronic cholecystitis, somatic symptom disorder, or LVNC at multiple institutions. A multidisciplinary team evaluation integrating cardiac imaging, technetium-99m phosphate scintigraphy, and genetic testing confirmed the diagnosis of ATTR-CA associated with the Ser23Asn (TTR c.128G>A p.Ser43Asn) variant. She was treated with tafamidis but died suddenly only two months after diagnosis. This case illustrates the multisystem involvement of ATTR-CA, in which atypical presentations frequently result in diagnostic delays. It represents a rarely reported instance of ATTR-CA presenting primarily with severe recurrent abdominal pain.

**Conclusion:**

In patients with ventricular hypertrophy, ATTR-CA may manifest with atypical symptoms such as severe, recurrent abdominal pain, which can result from autonomic neuropathy or gastrointestinal amyloid deposition. When the clinical diagnosis remains uncertain, multidisciplinary team consultation is essential to synthesize all available findings.

## Introduction

Transthyretin cardiac amyloidosis (ATTR-CA) is a progressive and fatal disease caused by the accumulation of misfolded proteins in the cardiac interstitium, resulting in restrictive cardiomyopathy and subsequent heart failure (1). Advances in non-invasive diagnostic techniques, particularly bone scintigraphy, have increased recognition of ATTR-CA (2). ATTR-CA exists in two primary forms: wild-type (ATTRwt) and hereditary (ATTRv). The hereditary form carries significant implications for genetic counseling and familial screening. Due to autonomic neuropathy, gastrointestinal manifestations are considerably more common in ATTRv than in ATTRwt (3). The clinical manifestations of ATTR-CA and left ventricular non-compaction (LVNC) are highly variable and non-specific, often leading to delayed or incorrect diagnosis, such as misclassification as hypertrophic cardiomyopathy (4). This diagnostic dilemma is further exacerbated in patients presenting with non-cardiac symptoms, such as chronic diarrhea or constipation. These symptoms may initially be attributed to more common gastrointestinal or psychiatric disorders, such as somatic symptom disorder, thereby masking the underlying cardiac pathology.

We report a case in which the clinical course was complicated by recurrent, severe abdominal pain. The patient was initially misdiagnosed with LVNC but was subsequently confirmed to have ATTR-CA.

## Case presentation

A 58-year-old woman presented to the emergency department with a five-month history of recurrent epigastric pain, accompanied by nausea and vomiting. The pain was intermittent but recurrent, characterized by periodic exacerbations of excruciating intensity. Prior to this admission, she had consulted multiple medical centers, but the etiology remained elusive and therapeutic interventions provided no relief. To identify the source of the pain and rule out acute abdominal pathology, contrast-enhanced computed tomography (CT) of the chest and abdomen was performed. The scan showed chronic cholecystitis, intraluminal gas distension of the gastrointestinal tract, ileocecal wall thickening, and omental and peritoneal thickening with blurring of the fat planes. However, these findings did not adequately explain the recurrent, severe abdominal pain. Retrospective review of previous medical records indicated that gastroscopy had revealed chronic non-atrophic gastritis, whereas colonoscopy findings were unremarkable. Echocardiography demonstrated left ventricular hypertrophy, yet she reported no history of hypertension, and cardiac magnetic resonance (CMR) imaging had previously suggested LVNC. Two months earlier, she had been diagnosed with somatization disorder and anxiety-depression. Given the clinical complexity, she was admitted to the gastroenterology department for comprehensive evaluation.

On physical examination, she weighed 40 kg and was 145 cm tall. Her blood pressure was 92/60 mmHg and her heart rate was 70 beats per min. Cardiac examination revealed leftward displacement of the cardiac border without pathological murmurs. Abdominal palpation elicited tenderness and rebound tenderness in the upper and middle quadrants. She also had mild, symmetrical pitting edema in both lower extremities.

Laboratory investigations revealed anemia (hemoglobin 103 g/L), while liver enzymes and lactate dehydrogenase levels remained within normal limits. Based on these findings, the initial diagnosis was incomplete intestinal obstruction or enteritis. However, laboratory tests revealed elevated myocardial injury markers (troponin T, 0.089 μg/L; reference range, 0–0.014 μg/L) and a markedly elevated N-terminal pro-B-type natriuretic peptide level (8,860 pg/mL; reference range, 0–220 pg/mL).

**Figure 1:**
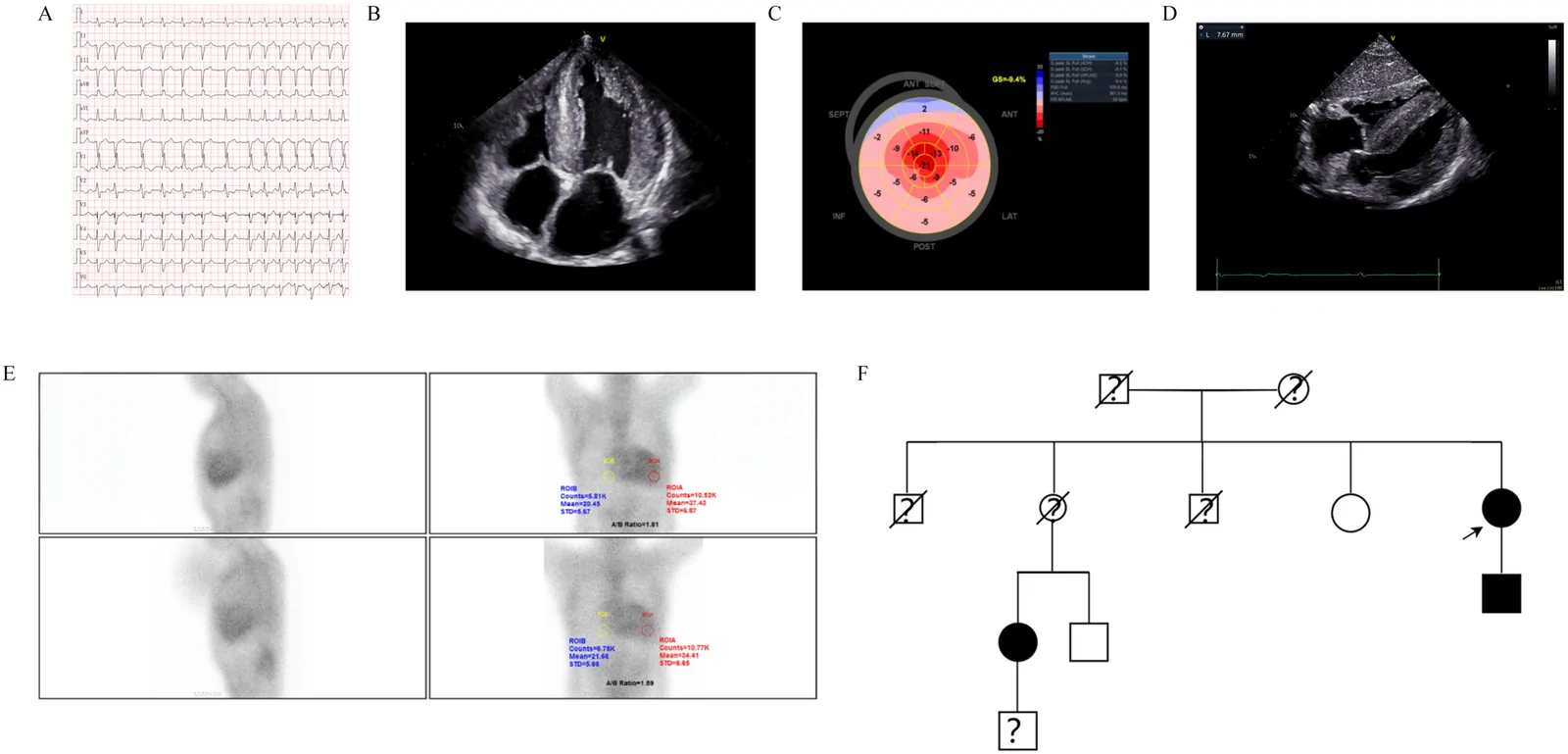
Imaging findings and family pedigree. Electrocardiography showed atrial flutter with variable conduction (2:1–3:1), complete right bundle branch block, left anterior fascicular block, abnormal *Q* waves in anterior leads **(A)**. Echocardiography showed ventricular hypertrophy in the four-chamber view **(B)**, an apical sparing pattern in the strain analysis **(C)**, and right ventricular wall thickening during diastole in the subxiphoid view **(D)**. The ^99^mTc-PYP scintigraphy showed Perugini Grade 3,characterized by diffuse myocardial uptake markedly exceeding activity in the adjacent ribs and sternum **(E)**. Family pedigree showed proband's niece and son were also diagnosed **(F)**. Arrow: the proband. Question mark: we were unable to determine whether that individual was involved.

Electrocardiography demonstrated complete right bundle branch block, left anterior fascicular block, abnormal *Q* waves in the anterior leads, and rightward cardiac rotation. Echocardiographic evaluation identified left atrial enlargement, left ventricular wall thickening (interventricular septal thickness: 15 mm; left ventricular posterior wall thickness: 13 mm), right ventricular wall thickening (right ventricular anterior wall thickness: 7.67 mm), and impaired systolic function (left ventricular ejection fraction: 41%); most notably, global longitudinal strain (GLS) was −9.4%, the quantitative apical sparing ratio was 1.1, and strain analysis revealed an apical sparing pattern (Figures 1B–D).

A repeat contrast-enhanced abdominal CT revealed no gallbladder wall thickening or inflammation, although bile stasis was evident. On the fourth hospital day, the patient's symptoms had not resolved and the underlying etiology remained unclear. A multidisciplinary team (MDT) comprising specialists in neuroradiology, neurology, cardiology, infectious diseases, psychiatry, and nutrition convened to evaluate the case. The initial working diagnosis favored LVNC complicated by heart failure; however, other causes of cardiac hypertrophy, particularly amyloidosis, required rigorous exclusion. Although the abdominal discomfort was provisionally attributed to psychogenic factors, additional investigations were undertaken to rule out atypical pathogens, heavy metal toxicity, and other organic etiologies.

Although the interferon gamma-induced protein 10 test indicated tuberculosis infection, Mycobacterium tuberculosis was not identified. Empirical anti-tuberculosis therapy was initiated after discussion with the patient's family, but the abdominal pain improved only minimally. Serum and urine free light chain levels were within normal limits, and immunofixation electrophoresis detected no significant abnormal immunoglobulins. Therefore, ATTR-CA was suspected. The patient underwent ^99^mTc-pyrophosphate (PYP) scintigraphy, which showed a heart-to-contralateral (H/CL) ratio of 1.81 at 1 h and 1.59 at 3 h, with a Perugini grade of 3 (Figure 1E). Subsequent genetic testing detected a transthyretin (TTR) gene variant, Ser23Asn (TTR c.128G>A p.Ser43Asn). The patient was ultimately diagnosed with ATTRv cardiac amyloidosis.

**Figure 2:**
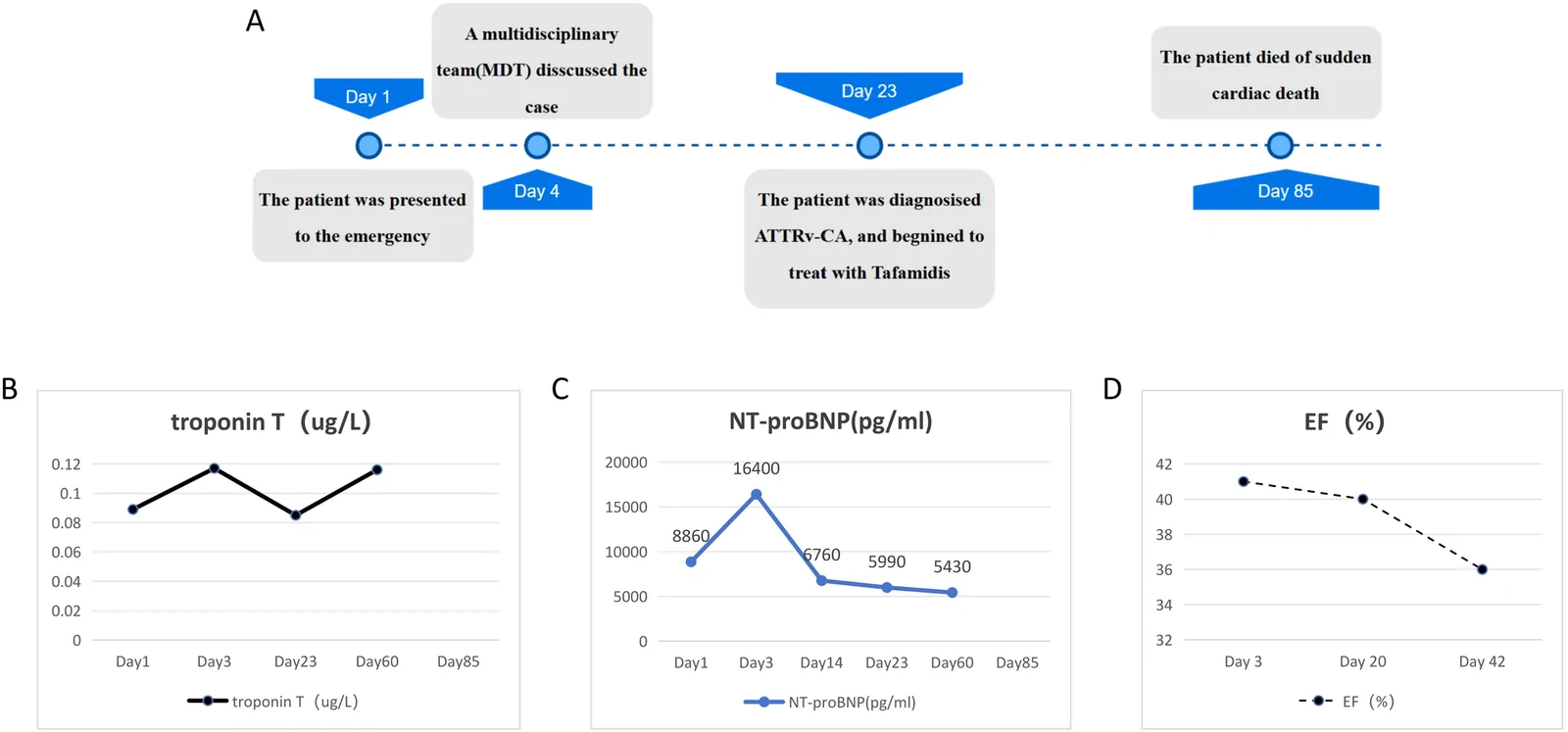
Treatment and outcome. The progression of her medical history following her first visit to our hospital **(A)**. Troponin T changed in the progression **(B)**. NT-proBNP changed in the progression **(C)**. EF changed in the progression **(D)**. EF, ejection fraction.

Treatment with tafamidis (61 mg once daily) was initiated; however, the patient continued to experience recurrent abdominal pain. She declined a proposed abdominal wall biopsy. Figure 2A shows the progression of her medical history following her first visit to our hospital. Holter monitoring on day 53 recorded 1,547 episodes of paroxysmal ventricular tachycardia, the longest of which lasted five beats; on day 60, she developed syncope, and electrocardiography confirmed atrial flutter with 2:1–3:1 conduction (Figure 1A), prompting initiation of anticoagulation. Although the patient continued medical therapy, her abdominal pain persisted. After two months of treatment, she experienced sudden cardiac death. During the treatment period, troponin T levels remained relatively stable (Figure 2B), while NT-proBNP levels showed a slight decline (Figure 2C) and ejection fraction (EF) decreased (Figure 2D).

Following the proband's diagnosis, her niece and son were also diagnosed. Her niece presented with palpitations, exertional dyspnea, left ventricular hypertrophy, and elevated levels of NT-proBNP and troponin T. Her son currently has dizziness, left ventricular hypertrophy, and reduced exercise tolerance. Figure 1F shows the familial pedigree.

## Discussion

ATTR-CA is a systemic disease, its clinical manifestations are heterogeneous and atypical, causing a significant delay in diagnosis. Cardiac symptoms such as heart failure and arrhythmia are the main manifestations of ATTR-CA, while digestive tract symptoms are also recognized although often overlooked. A case report describes an 88-year-old female whose initial symptoms were progressive abdominal pain and rectal bleeding, and was subsequently diagnosed with ATTRv (Val122Ile variant) (5). An abstract reported a case of ATTR amyloidosis complicated by intestinal hyperoxaluria and subsequent acute oxalate nephropathy resulting from autonomic neuropathy, presenting with altered bowel habits associated with abdominal pain (6). However, here the patient's main symptoms manifested as recurrent, episodic abdominal pain, representing a distinct and highly unusual clinical pattern. Furthermore, ATTR-CA has been reported to be associated with autonomic nerve dysfunction. In these patients, the most prominent symptoms are orthostatic hypotension, diarrhea, constipation, and early satiety, or excessive sweating, rather than recurrent severe paroxysmal abdominal pain (7). The patient experienced recurrent episodes of unbearable abdominal pain, which led clinicians to consider gastrointestinal disorders. Multiple abdominal CT examinations were conducted. At the same time, because the patient has not received a proper diagnosis for an extended period, her symptoms have not improved. This diagnostic delay has ultimately led to the development of psychological disorders. The cardiac magnetic resonance scan in the external hospital indicated LVNC, and the image quality was poor and no specific descriptions of T1 and ECV were provided, thus delayed the diagnosis. As shown in Table 1, the imaging characteristics of LVNC and cardiac amyloidosis differ significantly, particularly on CMR.While distinct CMR imaging features characterize cardiac amyloidosis and LVNC, CMR image quality may be degraded by arrhythmias, respiratory motion, and partial volume effects (8), thereby potentially confounding the diagnosis.

**Table 1:** Imaging features for differentiating LVNC from cardiac amyloidosis.

Types of imaging	LVNC	ATTR-CA
Echocardiography	End-diastolic NC (non-compacted)/C (compacted) ratio ≥2 >3 Apical trabeculations distal to the papillary muscles Intertrabecular recesses perfused from the LV cavity Trabeculations isoechoic to myocardium with synchronous contraction (16, 17)	Unexplained LV thickness (≥12 mm), diagnosis requires at least one of 1 or 2 ≥2 of 3 echocardiographic features: grade ≥2 diastolic dysfunction, tissue Doppler *s*′/*e*′/*a*′ < 5 cm/s, or GLS > −15% (absolute) Multiparametric score ≥8: RWT >0.6 (3 pts), *E*/*e*′ > 11 (1 pt), TAPSE ≤19 mm (2 pts), GLS ≤−13% (1 pt), apical/basal strain ratio >2.9 (3 pts) (1)
CMR imaging	Total trabecular mass compared to total LV mass NC ventricular mass > 20% of total LV mass (17)	Required (both): diffuse subendocardial/transmural LGE and abnormal gadolinium kinetics. Supportive: ECV ≥0.40%—strongly suggestive, but not essential/diagnostic (1).
Bone scintigraphy	Specific radionuclide imaging is generally not used for diagnosis.	PYP, DPD or HMDP scintigraphy shows Grade 2–3 myocardial uptake (1)
CT scan	NC/C ratio > 2.2 at end diastole seen in >2 segments (17, 18)	The left ventricular wall thickness, not specific

LVNC, left ventricular non-compaction; ATTR-CA, transthyretin cardiac amyloidosis; NC/C, non-compacted/compacted; LV, left ventricular; GLS, Global Longitudinal Strain; RWT, Relative Wall Thickness; pt, point; TAPSE, tricuspid annular plane systolic excursion; LGE, late gadolinium enhancement; ECV, extracellular volume; PYP, ^99^mTc-pyrophosphate; DPD: ^99^mTc-3,3-diphosphono-1,2-propanodicarboxylic acid; HMDP: ^99^mTc-hydroxymethylene diphosphonate.

Therefore, a MDT comprising specialists from cardiology, gastroenterology, neurology, radiology, and hematology is crucial (9). Through multidisciplinary discussion, seemingly disparate symptoms can be correlated. For cardiac amyloidosis (CA), a comprehensive assessment involving electrocardiogram, cardiac ultrasound, PYP imaging, and CMR imaging is required. Multimodality cardiac imaging has facilitated the early diagnosis of CA (10). This case highlights that when standard workups for abdominal pain yield no definitive cause, particularly alongside subtle electrocardiographic or echocardiographic abnormalities, CA must be actively ruled out.

Positive bone scintigraphy is characteristic of ATTR-CA; however, it is rarely observed in AL-CA cases (11). Positron emission tomography (PET) is emerging as a key diagnostic tool for cardiac amyloidosis (12). Multiple PET tracers, including 18F-florbetapir, 18F-florbetaben, 18F-flutemetamol, and 11C-Pittsburgh B (11C-PiB), have been successfully applied. By exploiting differences in time-dependent uptake kinetics, 18F-florbetaben enables differentiation between ATTR-CA and AL-CA on delayed imaging: AL-CA shows sustained high uptake, while ATTR-CA exhibits rapid washout (13). However, these tracers remain confined to research settings and have not been adopted in clinical practice.

In this case, formal autonomic function testing, histopathological confirmation of amyloid deposition, and gastrointestinal biopsy results were unavailable. Therefore, the proposed mechanisms remain speculative and should be regarded as hypothetical. First, amyloid proteins may deposit in the gastrointestinal tract. These nonspecific gastrointestinal symptoms often lead to a delay in diagnosis (14, 15). Second, patients with ATTR-CA may develop autonomic neuropathy, which can present as abnormal gastrointestinal motility (6). One possible mechanism is amyloidosis-associated cholecystitis, in which amyloid proteins may infiltrate the gallbladder wall and its microvessels (26).

Notably, genetic analysis identified a p.Ser43Asn mutation, differing from the predominant p.Val50Met variant reported in Northern China and the p.Ala117Ser variant common in Southern China (25). In China, p.Ser43Asn was first reported in the Western Han Chinese population (21). In fact, mutations at this TTR locus have not been limited to Chinese individuals; they have also been reported in ATTR-CA patients from Italy, Ecuador, and Colombia (Table 2). The patient exhibited symptoms of mental illness, thereby complicating the diagnostic process. This medical history may initially detract attention from the comprehensive assessment of the heart; this phenomenon is documented in functional neurological disorders but is rarely described in amyloidosis (27).

**Table 2:** Summary of TTR mutations located at p.Ser43Asn in population with hereditary ATTR-CA.

Author (year)	Involved cases	Age (years)	Sex (Male)	Cardiac	Neurologic	Mixed	Residence	Clinical outcome
Ihse (2013) (19)	2	44,44	1	2	0	0	Italy	/
Rapezzi (2012) (20)	2	/	/	1	0	1	Italy	/
Yu (2024) (21)	3	54,55,56	0	3	0	0	China	/
Papathanasiou (2020) (22)	3	44,45,53	1	3	0	0	Italy	1 died 2 years after diagnosis
Abulikemu (2025) (23)	1	50	1	1	0	0	China	During treatment
Castellar-Leones (2024) (24)	25	37.76	14	/	/	/	Ecuador and Colombia	/
Chu (2025) (25)	3	/	/	/	/	/	China	/

TTR, transthyretin; ATTR-CA, transthyretin cardiac amyloidosis.

Although the patient was treated with tafamidis, she died suddenly only two months after her diagnosis. Tafamidis acts by stabilizing the tetrameric form of transthyretin, and therefore cannot reverse amyloid deposits that have already formed. Therefore, cardiac stabilization with tafamidis primarily reflects the prevention of further myocardial infiltration. In contrast, gastrointestinal symptoms in ATTR amyloidosis are often secondary to autonomic neuropathy and direct infiltration of the enteric nervous system. The persistence of symptoms despite tafamidis therapy in our patient underscores that this agent should be regarded as a disease-modifying treatment aimed at halting further progression, rather than a symptomatic rescue therapy for established autonomic dysfunction. Future management for such patients may require a multidisciplinary approach, combining TTR stabilizers with symptomatic management for autonomic failure. Although sudden cardiac death (SCD) is more frequent in AL-CA than in ATTR-CA and its underlying mechanism is often unidentified, our patient had experienced paroxysmal ventricular tachycardia, paroxysmal atrial flutter, and a rapidly declining ejection fraction. These factors likely contributed to her SCD (28). However, unlike ischemic heart disease, SCD in ATTR-CA is frequently driven by pulseless electrical activity rather than ventricular tachyarrhythmias. Consequently, the utility of ICDs for primary prevention remains controversial, as they may not mitigate the risk of non-arrhythmic death (29). Our case illustrates the clinical dilemma of managing high-risk patients where the substrate for sudden death is deeply rooted in the diffuse, progressive nature of amyloid infiltration.

**Figure 3:**
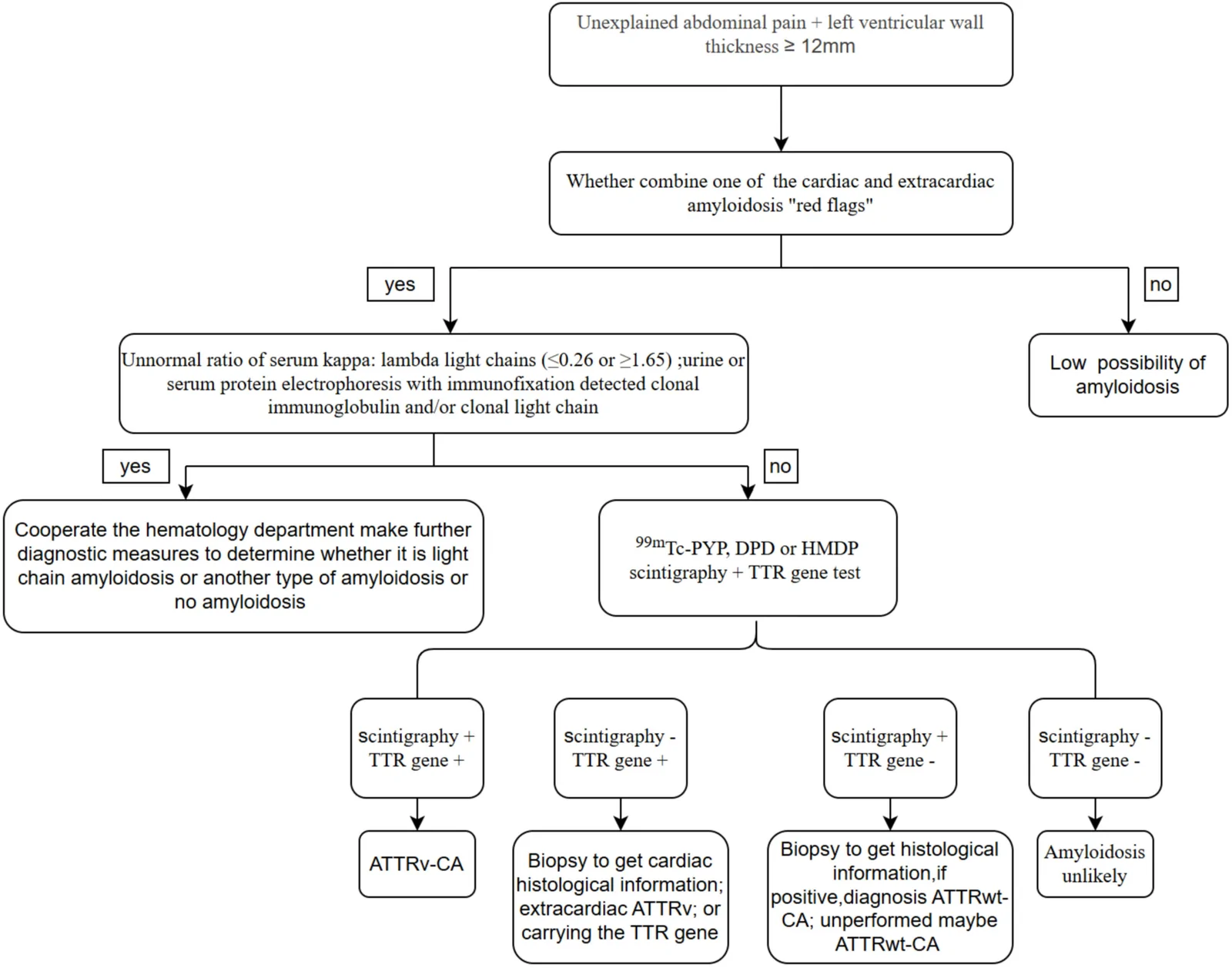
Diagnostic algorithm. Red flags (1): heart failure in ≥65years; aortic stenosis in ≥65years; hypotension or normotensive if previously hypertensive; sensory involvement, autonomic dysfunction; peripheral polyneuropathy;proteinuria; skin bruising; bilateral carpal tunnel syndrome; ruptured biceps tendon; subendocardial/transmural LGE or increased ECV; reduced longitudinal strain with apical sparing; decreased QRS voltage to mass ratio; Pseudo *Q* waves on ECG; AV conduction disease; possible family history.

This case underscores an important clinical lesson: in patients presenting with recurrent or severe localized abdominal pain and left ventricular hypertrophy, clinicians should maintain a broad differential diagnosis. If these patients also present with at least one of the cardiac amyloidosis “red flags,” screening for cardiac amyloidosis should be initiated. Figure 3 presents a diagnostic algorithm outlining the evaluation of patients with unexplained abdominal pain and left ventricular hypertrophy. As the algorithm indicates, multimodality imaging and genetic testing can establish the diagnosis.

Several limitations should be acknowledged. First, amyloid fibril deposition could not be histologically confirmed because consent for endomyocardial or abdominal wall biopsy was not obtained. Second, the neurological assessment was lacking, which may affect the strength of our conclusions, and future studies should incorporate these objective measures to validate these findings. Despite these constraints, this case provides valuable clinical insights. MDT consultation proved indispensable in managing this diagnostic challenge.

## Conclusion

Unexplained abdominal pain co-occurring with ventricular hypertrophy should prompt consideration of ATTR-CA. When diagnostic uncertainty remains, a MDT approach that integrates clinical manifestations and comprehensive imaging findings is essential for accurate differential diagnosis. Abdominal pain in ATTR may result from autonomic neuropathy or amyloid deposition in the gastrointestinal tract.

## Data Availability

The original contributions presented in the study are included in the article, further inquiries can be directed to the corresponding author.
